# Specific Phospholipids Regulate the Acquisition of Neuronal and Astroglial Identities in Post-Mitotic Cells

**DOI:** 10.1038/s41598-017-18700-4

**Published:** 2018-01-11

**Authors:** Aneley Montaner, Themis Taynah da Silva Santana, Timm Schroeder, Marcelo Einicker-Lamas, Javier Girardini, Marcos Romualdo Costa, Claudia Banchio

**Affiliations:** 1 Instituto de Biología Molecular y Celular de Rosario (IBR, CONICET) Ocampo y Esmeralda, Predio CONICET and Departamento de Ciencias Biológicas, Facultad de Ciencias Bioquímicas y Farmacéuticas, Universidad Nacional de Rosario, 2000, Rosario, Argentina; 2 Brain Institute, Federal University of Rio Grande do Norte, Av. Nascimento de Castro 2155, 59056-450 Natal, Brazil; 3 Department of Biosystems Science and Engineering, Cell Systems Dynamics, ETH Zurich, Basel, Switzerland; 4 Instituto de Biofísica Carlos Chagas Filho, Universidade Federal do Rio de Janeiro, 21949–902 Ilha do Fundão, Rio de Janeiro Brazil

**Keywords:** Cell biology, Neuroscience

## Abstract

Hitherto, the known mechanisms underpinning cell-fate specification act on neural progenitors, affecting their commitment to generate neuron or glial cells. Here, we show that particular phospholipids supplemented in the culture media modify the commitment of post-mitotic neural cells *in vitro*. Phosphatidylcholine (PtdCho)-enriched media enhances neuronal differentiation at the expense of astroglial and unspecified cells. Conversely, phosphatidylethanolamine (PtdEtn) enhances astroglial differentiation and accelerates astrocyte maturation. The ability of phospholipids to modify the fate of post-mitotic cells depends on its presence during a narrow time-window during cell differentiation and it is mediated by the selective activation of particular signaling pathways. While PtdCho-mediated effect on neuronal differentiation depends on cAMP-dependent kinase (PKA)/calcium responsive element binding protein (CREB), PtdEtn stimulates astrogliogenesis through the activation of the MEK/ERK signaling pathway. Collectively, our results provide an additional degree of plasticity in neural cell specification and further support the notion that cell differentiation is a reversible phenomenon. They also contribute to our understanding of neuronal and glial lineage specification in the central nervous system, opening up new avenues to retrieve neurogenic capacity in the brain.

## Introduction

Embryonic stem cell differentiation in specific cell lineages is a major issue in cell biology, and particularly, in regenerative medicine. Neural stem cells (NSCs) have the potential for self-renewal and differentiation into neurons, astrocytes and oligodendrocytes^1,2^. The balance between NSC self-renewal, generation of fate-committed progenitor cells and post-mitotic cells is tightly controlled by intrinsic signals^3^. In turn, environmental cues, through the activation of intrinsic pathways, activate different transcription factor networks leading to the specification of neuronal and glial progenitor cells, which in turn generate neurons and macroglial cells, respectively^4^. Yet, it remains unclear whether early differentiating post-mitotic cells could switch from neuronal to glial fates, and *vice-versa*. Astrocytes isolated from the postnatal cerebral cortex or cerebellum can be directly converted into neurons through the expression of single transcription factor (TFs)^5^. This lineage-conversion occurs, to a large extent, independently of cell division^5^, indicating that post-mitotic neural cell retain some degree of plasticity to switch lineage. However, it is unclear whether extrinsic signals could induce such changes in cell fate.

Despite the structural role of phospholipids as membrane building blocks, cellular membranes are rich in specialized phospholipids that act as signaling molecules *per se* or as reservoirs of lipid messengers which, in turn, regulate and interact with multiple other signaling cascades, contributing to development, differentiation, function, protection and cell repair^6–12^. We have previously demonstrated that cytidine triphosphate (CTP):phosphocholine cytidylyltransferase (CCTα) or choline kinase (CKα) overexpression enhances phosphatidylcholine (PtdCho) biosynthesis in neuroblastoma Neuro-2a cells and induces neuronal differentiation in the absence of retinoic acid^11^. This suggested role of PtdCho as a positive regulator of neuronal cell fate was further confirmed by demonstrating that lysophosphatidylcholine turns on neuronal differentiation by activation of the small G protein Ras and by triggering the Raf/MEK/ERK pathway^8^.

Here, we show that specific phospholipids play an important role in the fate-specification of post-mitotic neural cells *in vitro*. Exogenously supplemented PtdCho drives post-mitotic cells towards a neuronal lineage at the expense of astroglial cell-fate and by driving unspecified cells to neuronal fate. This effect depends on cAMP-dependent kinase (PKA) activation and works through the phosphorylation of the calcium responsive element binding protein (CREB). Conversely, phosphatidylethanolamine (PtdEtn) treatment is unable to affect neuronal differentiation, but promotes the acquisition of an astroglial fate in post-mitotic neural cells. Also different from PtdCho, PtdEtn activates the MEK-ERK pathway to promote astroglial differentiation. Collectively, our results reinforce the role of lipids as signaling molecules and provide evidence for the reversibility in neural cell specification during a limited time-window after cell-cycle exit.

## Results

### Phosphatidylcholine (PtdCho) and phosphatidylethanolamine (PtdEtn) enriches, respectively, neuron and astrocyte differentiation in culture

**Figure 1:**
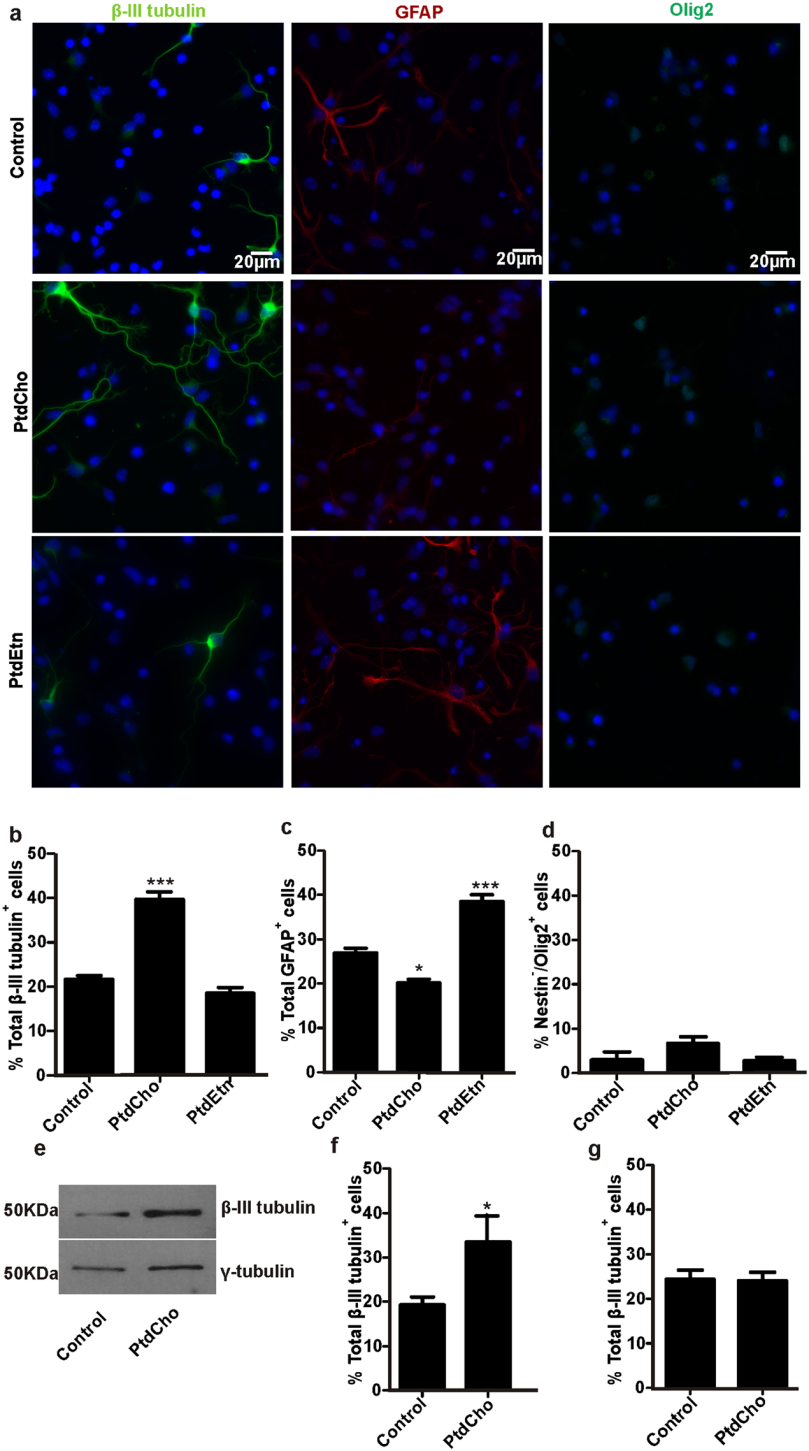
PtdCho and PtdEtn impact on neurosphere-derived cells differentiation. (**a**) Neurosphere-derived cells cultured during 3 days under differentiation condition (control) or in the presence of liposomes of PtdCho or PtdEtn were immunostained with antibodies against βIII-tubulin (green), glial fibrillary acid protein (GFAP) (red) or Olig-2 (green). Nuclei were counterstained with DAPI (blue). Pictures were taken with Nikon Model Eclipse 800 microscope and are representative of independent experiments conditions. (**b**–**d**) Graphs represent the percentage of neuronal (βIII-tubulin), astroglial (GFAP) and oligodendroglial (nuclear-Olig2) cells after 3 days under the indicated condition of differentiation. (**e**) Western blot analysis was performed for βIII-tubulin and γ-tubulin as a control. The gels/blots displayed here are cropped, and without high-contrast (overexposure). The full-length gels and blots are included in a Supplementary Information file. (**f**) Neurosphere-derived cells were treated with 50 μM of PtdCho for 1 hour and then the media was replaced for phospholipid-free media and incubated for 3 days. (**g**) Percentage of neuronal cells (βIII-tubulin positive cells) when PtdCho was added later on, after 24 h of culture and incubated for 3 days. Immunostained were performed after 3 days of culture in each assayed conditions. Graphs are representative of at least three independent experiments. Data were presented as mean ± SEM. ***p < *0*.*001*; *p < *0*.*05*.

PtdCho plays a critical role in driving the fate of neuroblast cells^11^. In order to address whether phospholipids could also impact on differentiation of neurons and macroglial cells from embryonic NSCs we used neurosphere-cultures from E13/14 dorsal telencephalon as a model. Neurospheres comprise mostly undifferentiated cells expressing Nestin, a cytoskeleton protein found mainly in neural progenitors (Supplementary Fig. 1). After dissociation and plating in medium without mitogens, neurosphere-derived cells differentiate into both macroglial (cells that express glial fibrillary acid (GFAP) or Olig-2) and neuronal (βIII-tubulin or MAP-2 positive) cells (Fig. 1a). Interestingly, addition of liposomes containing PtdCho (50 µM) or PtdEtn (50 µM) (both from eggs source) during differentiation affected the proportion of βIII-tubulin or GFAP expressing cells (Fig. 1a–c). Notably, we observed that treatment with PtdCho during the 3 days of differentiation significantly increased the proportion of βIII-tubulin positive cells (Fig. 1b and e) and decreased the number of cells expressing GFAP (Fig. 1c). Similar effects were observed after a short pulse (1 h) of PtdCho (Fig. 1f), suggesting that lipid treatment leads to cellular changes with long-lasting effects on cell specification. Supporting this, addition of PtdCho 1 day later after plating cells under differentiation condition did not promote neurogenesis (Fig. 1g), suggesting that the pro-neurogenic effects of PtdCho take place in the first stages of cell differentiation.

As free fatty acids play important roles as signaling molecules^13,14^, we evaluated the effect of sonicated liposomes of dioleyl-PtdCho (DO-PtdCho) and sphingomielyn on differentiation levels. We observed that all these lipids have the same effect on the rate of differentiation than the egg source-PtdCho, suggesting that the fatty acids composition is not crucial for the stimulus (see Supplementary Fig. 2).

In contrast, incubation with PtdEtn during 3 days increased the proportion of cells expressing GFAP (Fig. 1a and c) without affecting the rate of cells expressing βIII-tubulin (Fig. 1b). For both PtdCho and PtdEtn treatments we did not observe changes in the population of oligodendrocytes (Olig-2 positive cells) (Fig. 1a and d).

### PtdCho and PtdEtn do not affect neural progenitor cell proliferation *in vitro*

**Figure 2:**
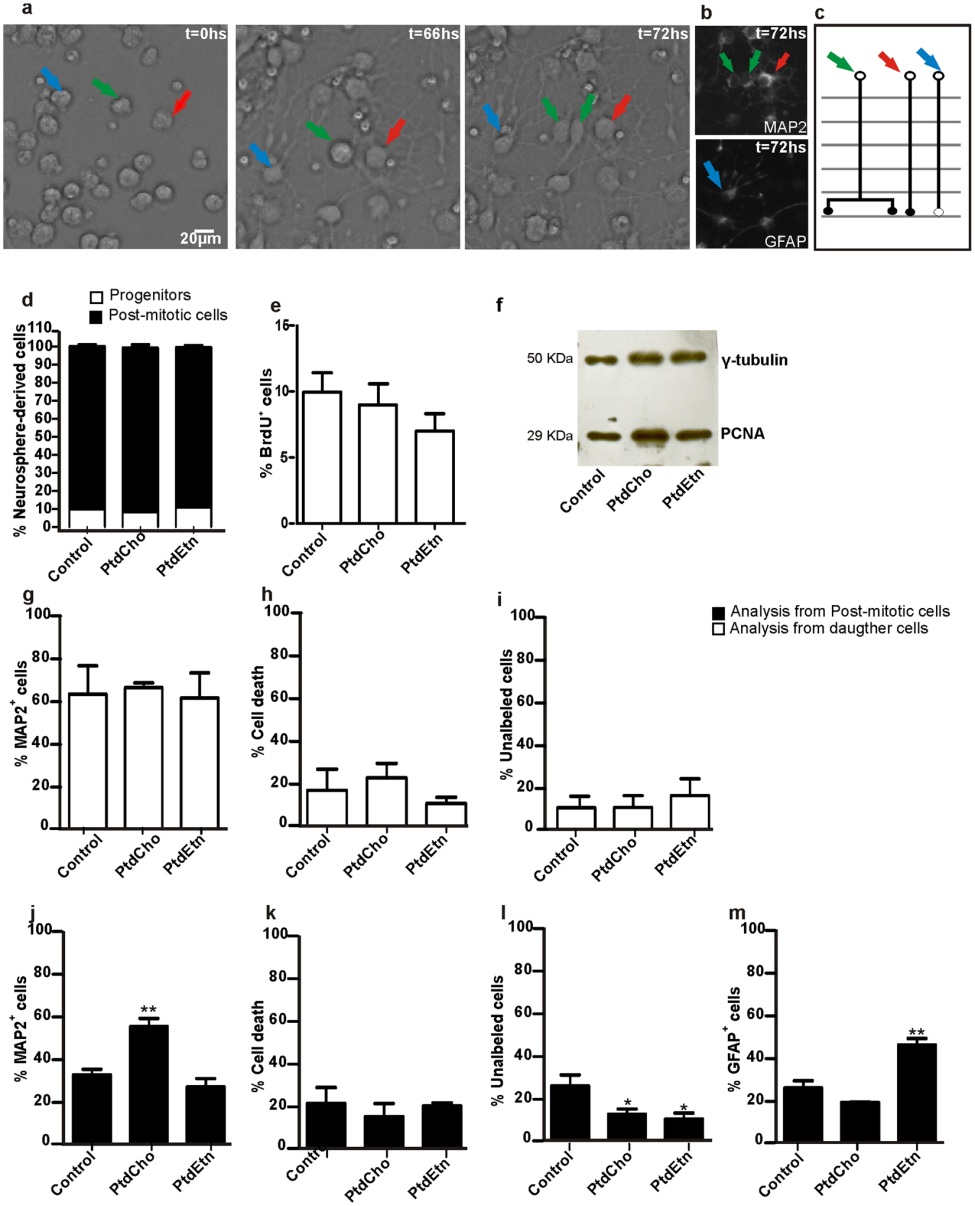
PtdCho and PtdEtn modified the fate of post-mitotic cells. (**a**) Bright field images of neurosphere-derived cells obtained by time-lapse video-microscopy. The numbers on the right upper corner indicate the time of imaging (See also Supplementary Movie). (**b**) Post-imaging immunocytochemistry of tracked cells after a 3-day experiment using antibodies against the neuronal protein MAP2 (upper panel) or the glial protein GFAP (lower panel). Observe that cells shown in panel (a) and indicated by green and red arrows express MAP2, whereas the cell pointed by a blue arrow expresses GFAP. (**c**) Schematic representation of lineage trees reconstructed from time-lapse recording. Green arrow: progenitor cellss that generate two daughter cells that express βIII-tubulin. Red arrow: post-mitotic cells that express βIII-tubulin. Blue arrow: post-mitotic cells that express GFAP. (**d**) Percentage of cells undergoing cell division (progenitors) or not (post-mitotic cells) during the 3-days period of real-time observation. (**e**) Quantification of BrdU labeled cells in each indicated condition. The analysis is representative of three independent experiments. (**f**) Western blot analysis was used to investigate the amount of PCNA and γ-tubulin as a loading control, in total extract obtained from neurosphere-derived cells cultured under the indicated conditions. (**g**) Quantification of MAP2+ neurons generated from progenitor cells or (**j**) differentiated from post-mitotic cells present in the culture since the beginning of imaging. (**m**) Quantification of GFAP+ astrocytes differentiated from post-mitotic cells. (**i**) Quantification of cells generated from progenitor cells during the 3-days imaging period expressing neither MAP2 nor GFAP (“unlabeled”). (**l**) Quantification of unlabeled non-dividing cells. (**h**) Quantification of cell death among cells generated from progenitor cells during the 3-days of observation. (**k**) Quantification of cell death among non-dividing cells. Data were presented as mean ± SEM *p < *0*.05; **p < *0*.*01*.

Since PtdCho is essential for cell proliferation^15,16^, we speculated that it could increase neurogenesis indirectly through the amplification of neuronal progenitors. To test whether the effects of PtdCho or PtdEtn treatment on the generation of neurons and astrocytes, respectively, could be due to an increased proliferation of fate-restricted progenitors, we tracked cell lineages of individual cells using live imaging. Neurosphere-derived cells were imaged up to 3 days using video time-lapse microscopy and analyzed using tTt v3.4.4^17^ (Fig. 2a and Supplementary movie). We observed that only 10% of neurosphere-derived cells underwent at least one round of cell division both under control and lipid treatment conditions (Fig. 2d), indicating that 90% of cells in our culture system have already left the cell cycle and became post-mitotic cells at the time of lipid treatments. These observations were further confirmed using BrdU-chasing (Fig. 2e). Only 10% of cells incorporated BrdU during the 3 days period of cell culture, indicating that a small fraction of neurosphere-derived cells are proliferative progenitors in all conditions examined. Further, we observed by Western Blot that the levels of the Proliferating Cell Nuclear Antigen (PCNA) were not affected by the addition of PtdCho or PtdEtn (Fig. 2f). Together, these observations suggest that effects of lipid treatments over progenitor cells are unlikely to explain our previous observations on neuronal and astroglial differentiation (Fig. 1).

Nevertheless, we analyzed the mode of cell division of progenitor cells, as this parameter can interfere with cell fate^18,19^. Cell divisions were classified in symmetric progenitor, asymmetric or symmetric terminal, based on the behavior of daughter cells. We observed that neither PtdCho nor PtdEtn treatment affected the rate of these different modes of cell division (Supplementary Fig. 3), further suggesting that the effect of lipid treatments on neural cell fate specification is independent of cell proliferation/division.

Furthermore, we analyzed the fate of the daughter-cells generated from the small set of progenitors undergoing cell division during the period of live imaging. To that, we performed post-imaging immunofluorescence analysis of tracked cells using antibodies against the neuronal marker MAP2 and the glial marker GFAP (Fig. 2b). As we mentioned before, only 10% of neurosphere-derived cells underwent at least one round of cell division in all the analyzed condition (Fig. 2d). From this population, we observed that about 60% of the daughter cells generated MAP2^+^ neuronal progeny (Fig. 2g), whereas 10% did give rise to cells labeling neither for GFAP nor for MAP2 (Fig. 2i). In addition, 20% of proliferating cells generated daughter cells that underwent cell death during the period of observation (Fig. 2h). Notably, we could not detect progenitor cells generating GFAP-expressing progeny during the 3 days of imaging. Both PtdCho and PtdEtn treatments did not significantly affect the fate of the dividing cells. Thus, the observed effect of those phospholipids on neural cell differentiation (Fig. 1) is independent of changes in progenitor behaviors.

Finally, we analyzed the fate of post-mitotic cells present in the cell culture since the beginning of the imaging period. To that, we sampled non-dividing neural cells in different fields of observation during the 3-days period of imaging and analyzed the fate by post-imaging immunofluorescence (Fig. 2b). We observed that a larger fraction of cells adopted a neuronal phenotype (MAP2^+^) in PtdCho-treated cultures, as compared to controls and PtdEtn-treated cultures (Fig. 2j). In contrast, PtdEtn enhanced astroglial differentiation (Fig. 2m). For both phospholipids, we also observed a reduction in the percentage of unlabeled (MAP2^−^/GFAP^−^) cells, suggesting that PtdCho and PtdEtn could encourage the acquisition of neuronal and astroglial fate, respectively (Fig. 2l). The frequency of cell death among non-dividing cells was unaffected by lipid treatments (Fig. 2k).

**Figure 3:**
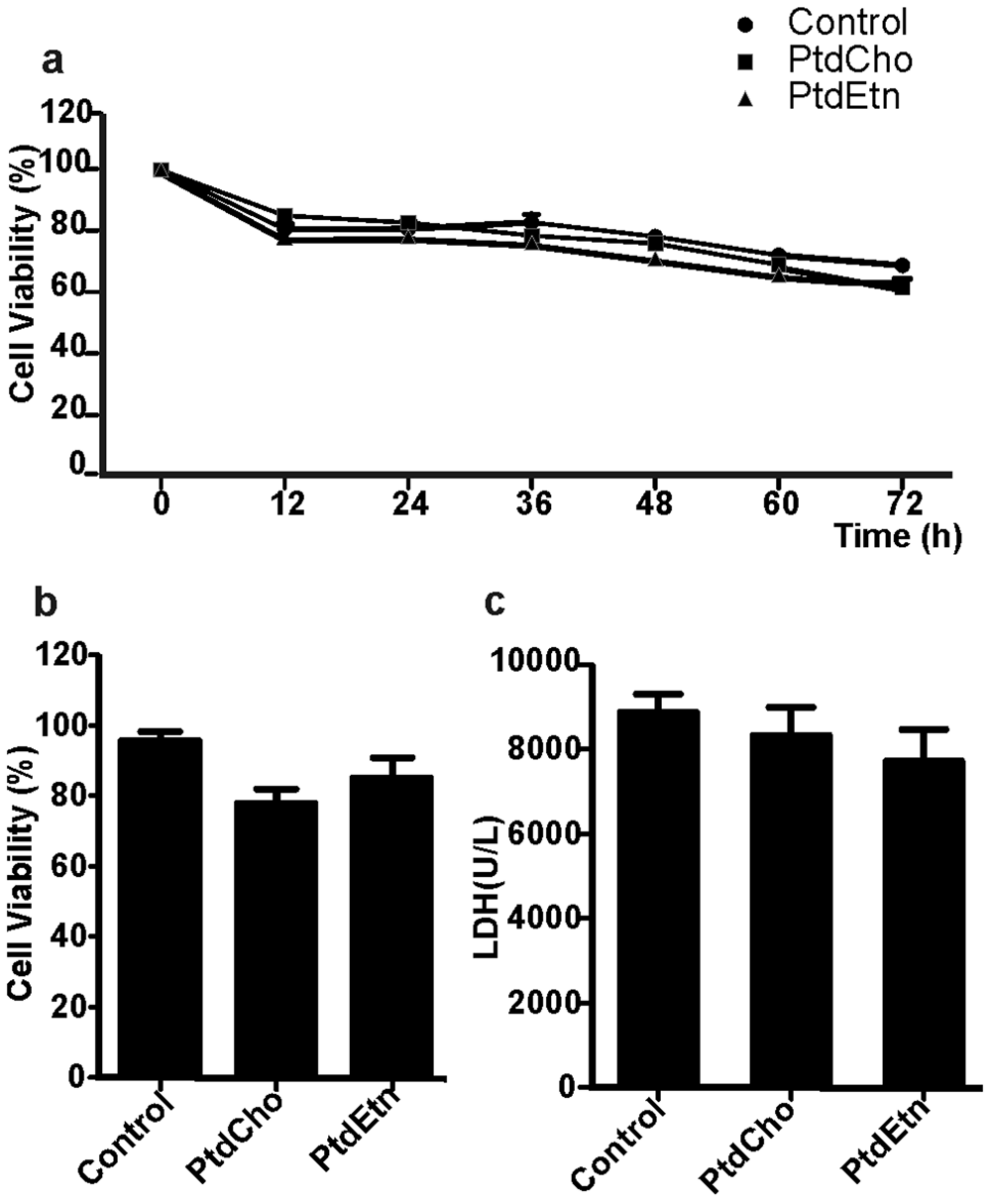
Cell viability is not affected by PtdCho or PtdEtn treatment. (**a**) Cell viability was assayed by counting the number of total live cells monitored by video time lapse microscopy every 12 h for each condition. The number of live cells at 12, 24, 36, 48, 60 and 72 h was divided by the total number of cells generated before these time-points within individual clones. (**b**) MTT assay after a 3-day experiment. (**c**) LDH assay after a 3-day experiment. Graphs are representative of three independent experiments. Data were presented as mean ± SEM.

Given the known role of lipids as neuroprotectors^10^, we next investigated whether cell survival of neurons and astrocytes could be selectively promoted by PtdCho and PtdEtn, respectively. To that, we monitored the total number of living cells per field of observation every 12 h using video time-lapse microscopy^20,21^. We did not detect any significant change in the frequency of cell death in cultures exposed to lipid treatments (3 days) as compared to controls (Figs 2h and k, and 3a). In accordance, we did not observe differences between lipids-treated and control cultures in MTT analysis^22^ and in the cytotoxicity assay measuring lactate dehydrogenase (LDH) activity (Fig. 3b and c) after a 3-day analysis. Altogether, these analyses indicate that lipid treatments do not significantly affect the survival of cells in our culture conditions.

### PtdCho and PtdEtn do not accelerate neuronal differentiation from neural post-mitotic cells

**Figure 4:**
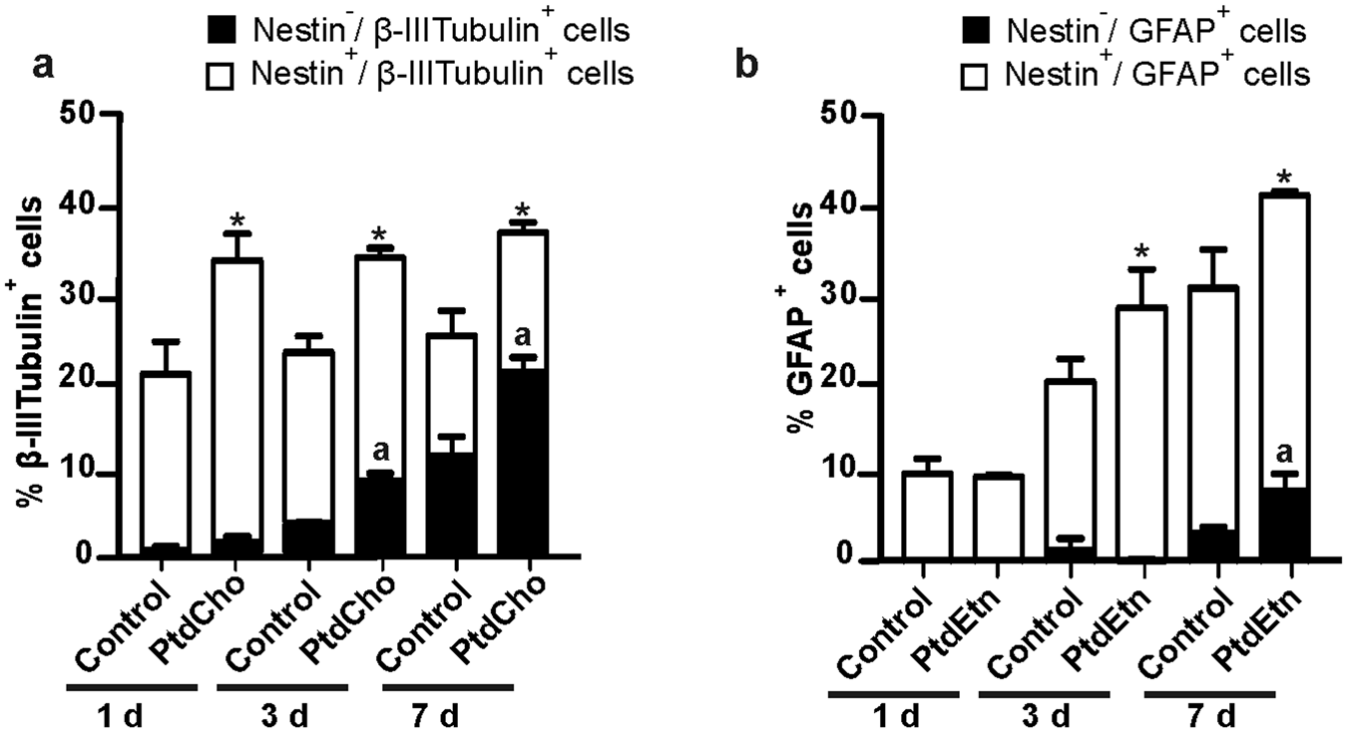
PtdCho and PtdEtn do not accelerate cell differentiation. (**a**) Percentage of Nestin positive/βIII-tubulin positive cells (white bars) and Nestin negative/βIII-tubulin positive cells (black bars) after 1, 3 and 7 days in culture under the indicated conditions. (**b**) Percentage of Nestin positive/GFAP positive cells (white bars) and Nestin negative/GFAP positive cells (black bars) after 1, 3 and 7 days in culture under the indicated conditions. Graphs are representative of three independent experiments. Data were presented as mean ± SEM. *p < *0*.*05;* a: *p* < *0*.*05*.

The increased differentiation of post-mitotic neural cells into neurons and astrocytes following treatment with PtdCho and PtdEtn could be explained by an acceleration of the differentiation process in the first 3 days of observation. To test this possibility, we quantified by immunofluorescence the percentage of Nestin/βIII-tubulin or Nestin/GFAP positive cells after 1, 3 and 7 days of lipids treatment (Fig. 4). We observed that already after 24 h, PtdCho treatment promoted a 1.8-fold increase in the percentage of cells expressing βIII-tubulin compared to controls. Most of these βIII-tubulin positive cells co-expressed Nestin, suggesting that they are early differentiating post-mitotic neurons that still retain some Nestin protein but have already up-regulated the expression of βIII-tubulin (Fig. 4a). According to this interpretation, the percentage of cells expressing only βIII-tubulin increased at 3 and 7 days, but the percentage of neurons in control conditions remained significantly lower than that in PtdCho-treated cultures (Fig. 4a). This result suggests that the increase in βIII-tubulin expressing cells caused by PtdCho is not due to the fastening of neuronal differentiation, but rather to a genuine increase in the number of cells adopting a neuronal phenotype (Fig. 4a). Similarly, we studied if PtdEtn could accelerate astrogliogenesis. The percentage of GFAP positive cells was about 10% in both control and PtdEtn at day 1, and virtually all cells co-express Nestin (Fig. 4b). At day 3, however, the frequency of GFAP positive cells in PtdEtn treated cultures increased and overcome the control. Interestingly, at day 7, we observed that the amount of GFAP/Nestin positive cells remained higher than the control, and that about 10% of GFAP cells lost Nestin expression in the PtdEtn group while remained constant in the control, suggesting that this lipid could also stimulate astrocyte maturation (Fig. 4b).

### PtdCho and PtdEtn modulate the acquisition of neuronal and astroglial fates, respectively

**Figure 5:**
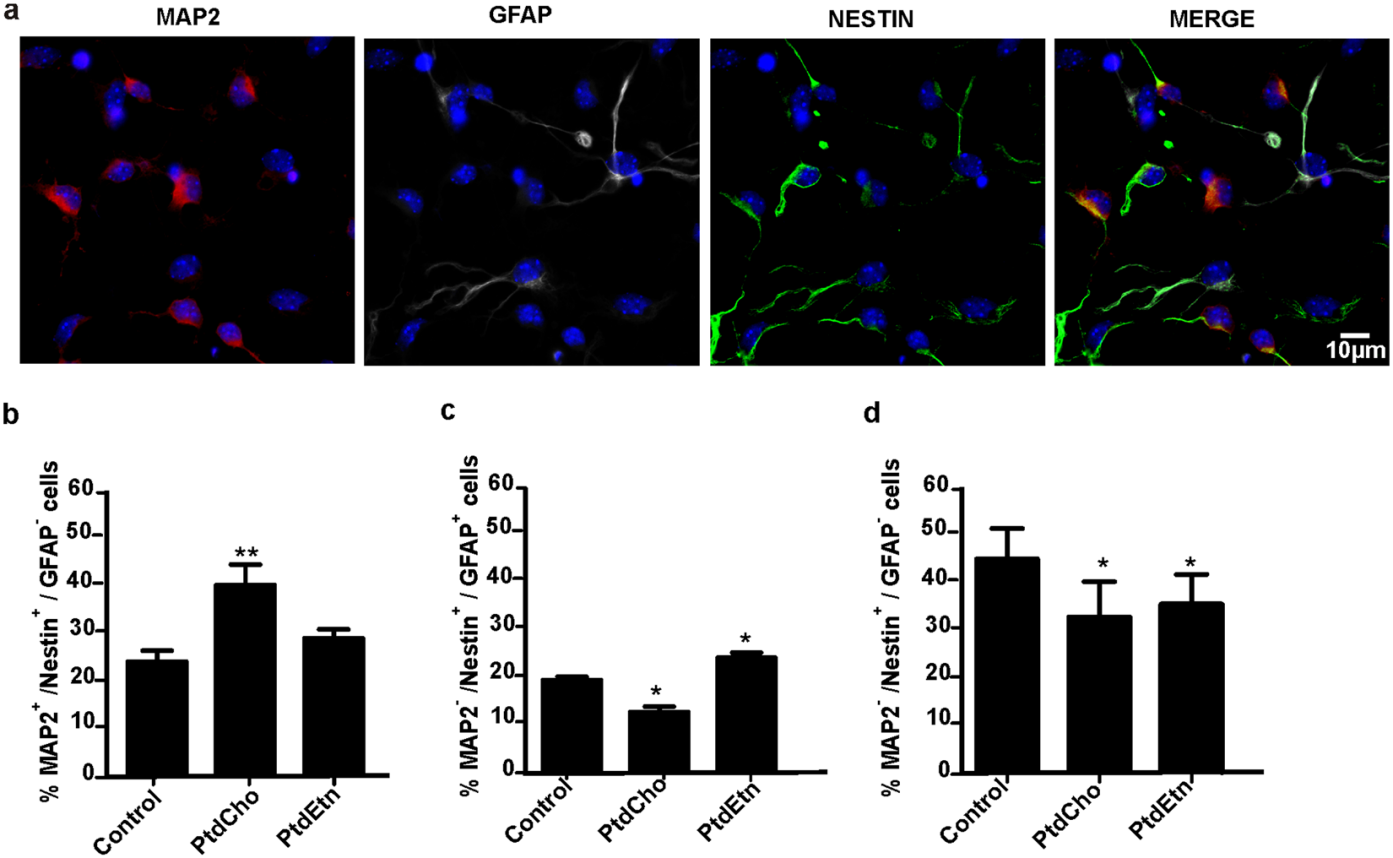
Phospholipids modulate the acquisition of neuronal and astroglial fates. Neurosphere derived-cells were incubated under differentiation condition plus PtdCho or PtdEtn for 3 days. (**a**) Representative images of cells stained with MAP2 (red), glial fibrillary acid protein (GFAP) (white), Nestin (green) and nuclei (DAPI) and visualized by confocal microscopy. The full pictures are included in a Supplementary Information file. (**b**) Percentage of neuronal-specified post-mitotic cells (Nestin positive/MAP2 positive/GFAP negative cells) after 3 days in culture. (**c**) Percentage of astrocyte-specified post-mitotic cells (Nestin positive/GFAP positive/MAP2 negative cells) after 3 days in culture. (**d**) Percentage of unspecified post-mitotic cells (Nestin positive/MAP2 negative/GFAP negative cells) after 3 days in culture. Data were presented as mean ± SEM *p < *0*.05; **p < *0*.*01*.

We hypothesized that PtdCho and PtdEtn could be acting in the initial phases of cell differentiation to instruct different neural cell phenotypes. To directly test this possibility, we quantified the percentage of cells expressing the neuronal marker MAP2, GFAP and Nestin (Fig. 5a). Again, we observed an increase in the amount of neuronal-specified cells (MAP2^+^/Nestin^+^) in cultures treated with PtdCho for 3 day as compared to controls (Fig. 5b). Interestingly, under the same condition, the number of astroglial-specified cells (GFAP^+^/Nestin^+^) and unspecified cells (Nestin^+^/GFAP^−^/MAP2^−^) was reduced after 3 days of incubation with PtdCho (Fig. 5c and d), suggesting that PtdCho-induced neuronal differentiation occurs at the expense of astrogliogenesis and by turning a population of unspecified cells to neuronal fate. Similar effects of PtdCho on neuronal differentiation were observed in primary cultures of E13 dorsal telencephalic cells (Supplementary Fig. 4), further supporting the pro-neurogenic role of that lipid. In contrast, the enhanced astroglial differentiation (Nestin^+^/GFAP^+^ cells) observed after PtdEtn treatment (Fig. 5c) was not accompanied by a decrease in the proportion of early differentiating neurons (Nestin^+^/MAP2^+^ cells) (Fig. 5b), but it led to a decrease in the percentage of unspecified cells (cell that only expressed Nestin) (Fig. 5d). Accordingly, when primary culture of E13 dorsal telencephalic cells (enriched in neuronal-specified cells) were incubated with PtdEtn, no GFAP positive cells were detected during 5 days of incubation reinforcing that PtdEtn raises astrogenesis without affecting neuronal differentiation.

Collectively, these results suggest that PtdCho modulates the acquisition of neuronal fate in detriment of astroglial ones, and driven unspecified cells to neuronal phenotype, whereas PtdEtn stimulates astroglial differentiation from uncommitted post-mitotic cells without affecting neurogenesis.

### PtdEtn but not PtdCho effects depend on the MEK-ERK pathway

**Figure 6:**
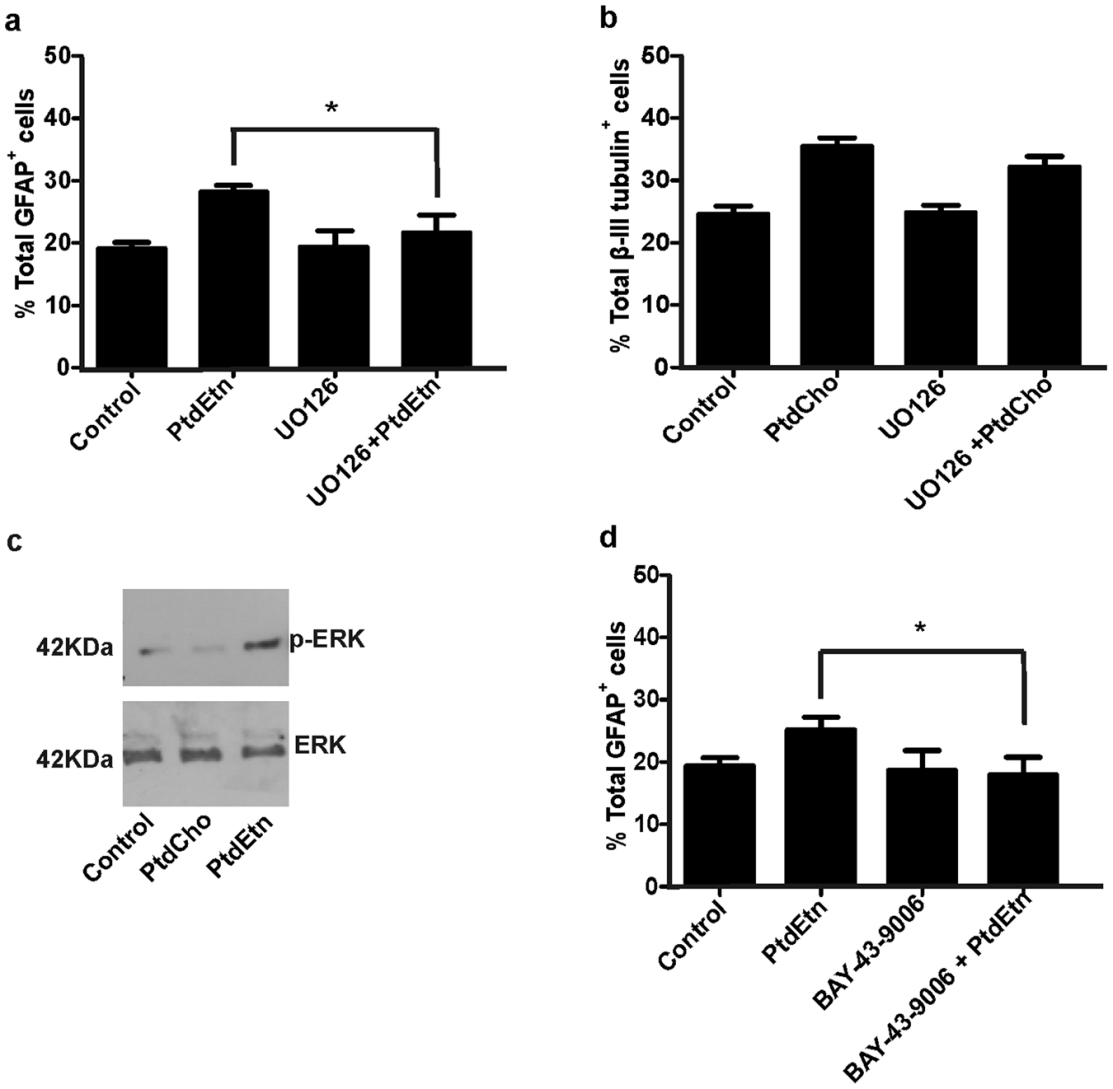
Astrocyte differentiation but not neuronal differentiation is affected by blocking the MEK pathway. (**a**) Graph represents the percentage of astrocyte differentiation in the presence and in the absence of the MEK inhibitor (U0126 (20 μM)) and PtdEtn. (**b**) Graph represents the percentage of neuronal differentiation in the presence and in the absence of MEK inhibitor (U0126 (20 μM)) and PtdCho. (**c**) Western blot analysis was used to investigate the amount of p-ERK and ERK (control) in cells treated with the indicated phospholipids or control cells. The gels/blots displayed here are cropped, and without high-contrast (overexposure). The full-length gels and blots are included in a Supplementary Information file. (**d**) Graph represents the percentage of neuronal differentiation in the presence and in the absence of the Raf inhibitor (BAY-43-9006 (3.5 μM)) and PtdEtn. Values were obtained from three independent experiments. Data were presented as mean ± SEM *p < *0*.*05*.

Previous studies have demonstrated that EGFR promotes astrocyte differentiation at late embryonic and neonatal stages of cortical development, in a process dependent on the EGFR/ERK signaling pathway^23^. As we demonstrated that PtdEtn promotes astrocyte differentiation, in order to identify the signaling pathway involved, we analyzed the effect of a MEK inhibitor U0126^24^ on this process. For these experiments, cells were seeded on lysine-treated plates for 2 h and then incubated in the presence or absence of lipids. When indicated, cells were incubated during 30 min with the MEK inhibitor U0126 (20 μM) prior to liposomes addition. Immunofluorescence was performed after 3 days of incubation. As Fig. 6a shows, U0126 treatment clearly decreased the frequency of astrocyte differentiation induced by PtdEtn without affecting basal glial differentiation (control condition). Moreover, U0126 did not affect neuronal differentiation (Fig. 6b). Reinforcing the role of MEK-ERK pathway in astroglial differentiation promoted by PtdEtn, we also demonstrated an increase in the levels of p-ERK in cell cultures treated with PtdEtn for 5 min, as compared to controls or PtdCho-treated conditions (Fig. 6c).

### PtdCho-induced neuronal differentiation depends on the PKA/CREB pathway

**Figure 7:**
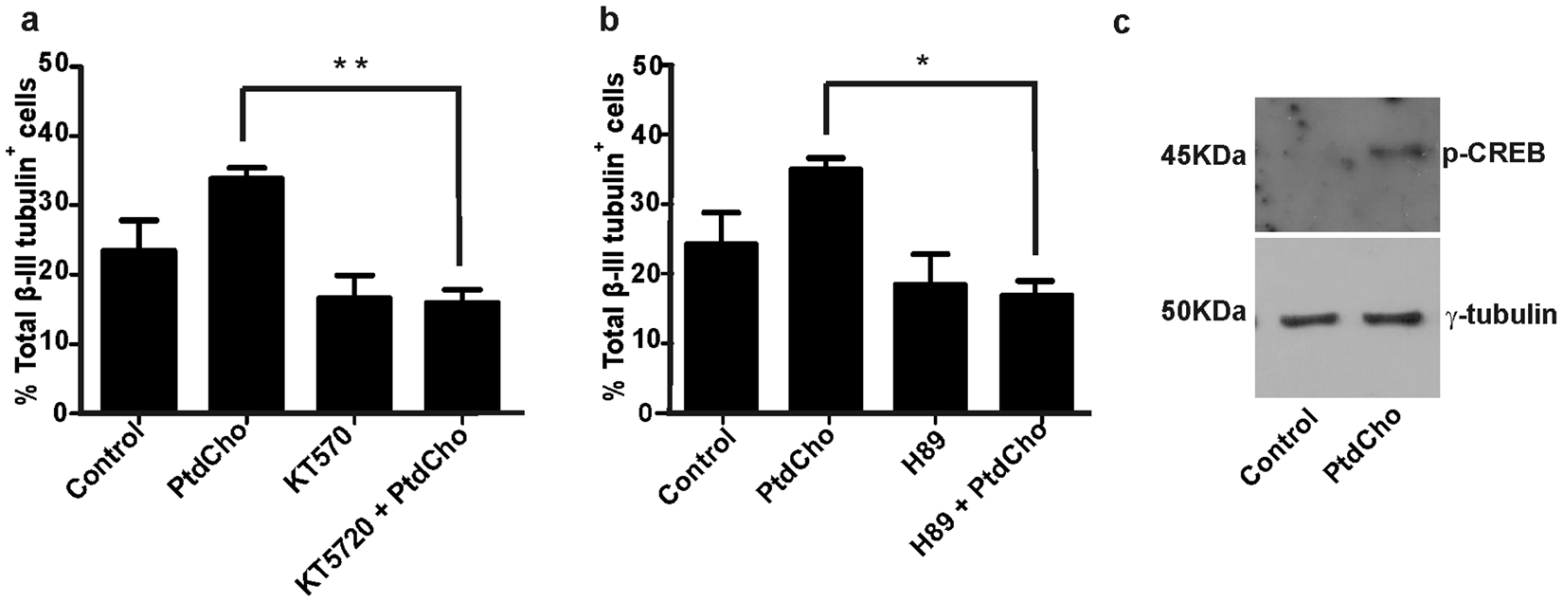
Neuronal differentiation is affected by blocking the PKA pathway. (**a**) Cells were cultured in media containing PtdCho in the presence or absence of the PKA inhibitor (KT5720 (10 μM)) and analyzed by immunofluorescence. Graph represents the percentage of neuronal differentiation measured in three independent experiments. (**b**) Cells were cultured in media containing PtdCho in the presence or absence of the PKA inhibitor (H89 (10 μM)). Graph represents the percentage of neuronal differentiation measured in two independent experiments. Data were presented as mean ± SEM *p < *0*.*05*, **p < *0*.*01*. (**c**) Western blot analysis was performed for p-CREB and γ-tubulin as a control. The gels/blots displayed here are cropped, and without high-contrast (overexposure). The full-length gels and blots are included in a Supplementary Information file.

Numerous studies have demonstrated the involvement of cAMP/PKA/CREB signaling pathway in neural differentiation^20,25,26^. In this sense, synaptamide, an endogenous metabolite of docosahexanoic acid, has a potent neurogenic activity via PKA/CREB phosphorylation. In addition, it was demonstrated that phosphorylation of CREB at Ser133 occurs in immature cortical neurons in early stages of neuronal differentiation^20^. To evaluate if PtdCho induced-neurogenesis depends on PKA signaling pathway, we evaluated the effect of two PKA inhibitors (KT5720 and H89). For this experiment, cells were seeded on lysine-treated plates for 2 h and then incubated in the presence or absence of lipids. When indicated, cells were incubated during 30 min with the PKA inhibitors prior to liposomes addition. Immunofluorescence was performed after 3 days of incubation. We observed that both inhibitors blocked PtdCho-induced neuronal differentiation (Fig. 7a and b). Considering the involvement of PKA/CREB in neuronal differentiation^20,27^, we next evaluated the levels of p-CREB in neural cells after 1 h of incubation under control or PtdCho-treated conditions. Total cellular extracts were analyzed by western blot using anti-p-CREB and anti-γ-tubulin (loading control) antibodies. Figure 7C shows that the levels of p-CREB clearly increased in cells treated with PtdCho. Thus, the pro-neurogenic effect of PtdCho is dependent of the activation of PKA/CREB signaling in early post-mitotic neural cells.

## Discussion

NSCs have the potential for self-renewal and, alternatively, for differentiation into neurons, astrocytes and oligodendrocytes. The balances between growth and differentiation and between glial and neuronal differentiation play a key role during brain development and, in particular, for brain regeneration after damages or injuries^28,29^. It is well known that the central nervous system (CNS) shows a modest recovery after injury due to the factors present in the wounded microenvironment that prevent neuronal differentiation and favor glia-scare formation. Thus, it is essential to generate a permissive microenvironment for NSCs and conduct them to differentiate towards functional neurons. However, little is known about the mechanism that regulates the commitment of NSCs^29^ and, in general, is considered an irreversible step in fate-determination during CNS development.

In this work, we have provided evidence that the fate of post-mitotic neural cells can still be changed by exogenous treatment with specific phospholipids. While PtdCho increases neuronal differentiation, PtdEtn enhances astroglial differentiation. Interestingly, PtdCho increases neuronal differentiation at the expense of astrocytes, suggesting that early post-mitotic cells are still not irrevocably committed towards a given phenotype. We also showed that PtdCho controls neuron specification through the activation of PKA/CREB, whereas PtdEtn stimulates astrocyte differentiation through the activation of the MEK/ERK signaling pathway. Altogether, our data shed new light on the understanding of neural cell specification and may contribute to the development of new strategies of enhancing neuronal differentiation in the injured adult CNS.

Neural progenitor fate-restriction is largely accepted as the main mechanisms underlying the generation of neuronal and macroglial cells during development^30,31^; and in the adult CNS^32,33^. As phospholipids play a key role for membrane biosynthesis and because its integrity is essential for cell division and survival^15,16^, we first speculated that phospholipids could selectively promote the expansion or survival of specific progenitor cells and consequently the generation of neuron and astrocytes. To our surprise, however, neither cell proliferation nor survival was affected by phospholipids treatments (Figs 2 and 3). Video time-lapse microscopy analysis showed that after plating neurosphere-derived cells in the absence of growth factors, only 10% of cells proliferate and, therefore, could be considered as progenitors (Fig. 2d and e). This percentage did not change with the presence of phospholipids in the media, indicating that the observed effect is not a consequence of an increase in proliferation of fate-restricted progenitors. Moreover, we also demonstrated that the fate of cells generated from the small population of progenitors in the culture is unchanged by phospholipid treatments (Fig. 2g–i), indicating that the effect of lipids on cell specification occurs on post-mitotic cells. Indeed, we could show that the fate of post-mitotic cells was affected by PtdCho and PtdEtn treatments, which enhanced neuronal and astroglial differentiation respectively (Fig. 2j and m). The finding that progenitor cells are unable to respond to lipids, like the post-mitotic cells do, provides new keys about the mechanism of cellular specification. We speculate that, similarly to cell reprogramming, this process is directly influenced by the cellular context (chromatin, proteosome or metabolome) and, perhaps, the different physiological states of those cells explain the different responses to lipids^34–36^.

A time course analysis of cell differentiation demonstrated that all along differentiation (1, 3 and 7 days) PtdCho-treated cultures always showed higher levels of neuron-specified cells (Fig. 4a). In the case of PtdEtn, however, treated-culture showed higher levels of astrocytes just after 3 days of incubation. The results suggest that these phospholipids do not simply speed up the early acquisition of post-mitotic cell fate, but rather have a genuine effect on cell fate acquisition. Accordingly, the percentage of more mature neurons (Nestin^−^) increased with time, but remained higher in PtdCho treated cells. In PtdEtn treated cultures, however, we observed a clear increase of GFAP positive cells after 7 days in culture, suggesting that besides affecting the acquisition of astroglial fate, PtdEtn also stimulates astrocyte maturation (Fig. 4b).

Commitment of stem cells to different lineages is regulated by many cues in the local tissue microenvironment^28^. After further examining the role of phospholipids in NSCs specification, we demonstrated that PtdCho and PtdEtn change the specification of post-mitotic neural cells (Fig. 5). In particular, PtdCho turns astroglial-specified cells and unspecified-cells to neural-specified cells (Fig. 5). Interestingly, the effect of PtdCho on neuronal specification is observed even after a brief exposure (1 h) to this lipid in the first day of culture (Fig. 1f). However, lipid treatment 24 h after plating the cells did not affect neuronal differentiation (Fig. 1g), indicating a narrow time-window of plasticity in post-mitotic cells. PtdEtn modified and turns a population of unspecified cells to astroglial cells without affecting the population of neuronal post-mitotic cells (Fig. 5).

The demonstration that a population of post-mitotic cells can become astrocytes or neurons without altering proliferation or cell death, provides direct evidence that specific phospholipids-mediated signals can modulate early stages of differentiation by regulating the specification of non-dividing neural cells. These observations indicate that neuronal and astroglial cell fates are not irreversibly determined at the progenitor cell stage, and that the final fate of post-mitotic cells could still be influenced by extrinsic cues.

Extracellular phospholipids usually exert their functions through G protein coupled receptors (GPCRs), which are linked to different protein kinases that linked-signaling pathway^6,8,37^. Here we show that PKA is required for PtdCho-induced neuronal differentiation of neurosphere-derived cells. Notably, inhibition of PKA completely abolished PtdCho-induced neuronal differentiation (Fig. 7a and b). However, it did not affect basal differentiation, which suggests that other signaling proteins besides PKA also contribute to the promotion of neuronal differentiation. Though the mechanism is yet unknown, PtdCho might directly or indirectly regulate the activity of an adenylate cyclase, thereby increasing cyclic AMP (cAMP) levels and activating the cAMP-dependent kinase (PKA). Numerous studies have indicated the involvement of PKA/CREB signaling pathway in neurosphere-derived cells differentiation^38,39^. According to our results, PKA/CREB signaling is involved in the PtdCho-induced neuronal differentiation. We have demonstrated that PtdCho induces CREB phosphorylation (Fig. 7c), and thus, by the activation of this transcription factor could regulate the expression of many target genes such as NeuroD, an early neurogenic transcription factor^27,40,41^.

We have also demonstrated that PtdEtn activates the MEK/ERK pathway, being an essential step for the stimulation of astroglial differentiation (Fig. 6). A possible explanation is based on the role of RKIP, a member of the PEBP (PtdEtn binding protein), as a negative regulator of Raf-1 and MEK^42–44^. Perhaps the binding of PtdEtn to RKIP might induce conformational changes that disrupt its interaction with Raf and MEK, leading to ERK activation and thus, astroglial differentiation^42,23,45^. Favoring this hypothesis, the effect of PtdEtn on glial differentiation was diminished by incubation with the Raf inhibitor BAY-43-9006 (3.5 μM) (Fig. 6d). In addition, it was demonstrated that the hippocampal cholinergic neurostimulating precursor protein (HCNP-pp) also a PEBP, regulates cell proliferation and differentiation by modifying the MAPK cascade. In fact, the levels of HCNP-pp regulate the fate of adult rat hippocampal cells, but different to our results, affecting progenitor cells^46,47^.

In this scenario, a repeated question arises: where do these phospholipids come from? Many populations of cells, in addition to astrocytes and neurons, are essential for brain development and function. Thus, it is no surprise that each type of cell could modulate or control the function, fitness or even the behavior of its counterparts, which implies a cross talk between cells^48^. A novel mechanism of cell-cell communication involves exosomes and, perhaps, they are the source of the phospholipids involved in the described effect^49,50^. Highlighting that phospholipids are not only the building support of these vesicles but those lipids themselves or their derivative-metabolites, could also carry a biological message. Supporting this notion, it was previously described that astrocyte-derived phosphatidic acid promotes dendritic branching^7^. Future investigation will focus in this hypothesis.

## Methods

### Chemicals and antibodies

Dulbecco’s modified medium/Ham’s F12 (DMEM/F12 1:1), B27, anti-rabbit Alexa Fluor® 488-labeled, anti-rabbit Alexa Fluor® 633-labeled and anti-chicken Alexa Fluor® 488-labeled were purchased from Life Technologies Corporation (Carlsbad, CA, USA); epidermal growth factor (EGF), human basic fibroblast growth factor (bFGF), protease inhibitor cocktail, poly-D-lysine (PDL), mouse anti-MAP2 and fetal bovine serum from Internegocios (Buenos Aires, Argentina). Rabbit anti-glial fibrillary acidic protein (GFAP) was purchased from DAKO (Carpinteria, CA, USA); rabbit anti-β-Tubulin III antibody from Sigma (St. Louis, MO, USA), rabbit anti-ERK and rabbit anti-pERK antibodies from Cell Signaling Technology (Beverly, MA, USA); rabbit anti-Olig2 and anti-mouse Cy3-labeled from Millipore (Massachusetts, USA). Rabbit anti-p-CREB was purchased from Santa Cruz (Dallas, Texas, USA) and chicken anti-Nestin from Abcam (Cambridge, UK). The inhibitors used were U0126 (Promega)^8^, H89 and KT5720 (Santa Cruz (Dallas, Texas, USA)^51^ and BAY-43-9006 (Cayman, Michigan, USA)^8^.

Phosphatidylcholine (P3556) and phosphatidylethanolamine (P7943) from egg yolk source were from Sigma (St. Louis, MO, USA). As specified in product information, they have a purity over 99% and a fatty acid content of approximately 33% palmitic, 13% stearic, 31% oleic, and 15% linoleic. In addition the detailed fatty acid composition of the mixture of egg yolk phosphatidylcholine and phosphatidylethanolamine has been recently described^52,53^.

### Animals studies and fetal neural stem cell culture

All animal experiments and related experimental protocol were approved by the Bioethics Commission for the Management and Use of Laboratory Animals from National University of Rosario, Argentina (N 6060/89). The methods were carried out in accordance with the approved guidelines (Guide for the care and use of Laboratory Animals- 8° edition- The National Academies press-Washington DC 2011 and Guidelines on: procurement of animals used in science. Canadian Council on Animal Care). Time pregnant female C57/BL6 mice (gestation day 13) were sacrificed under supervision of the Animal Care and Use Committee. Neurospheres were obtained from E13 cortical cells as previously described^54^. Briefly, the lateral portion of the dorsal telencephalon (cortex) of embryonic day 13 mouse C57/BL6 was isolated. The cortices were first chemically disrupted adding tripsine (0.05% w/v) for 5 minutes and then mechanically disrupted into single cells by repeated pipetting in medium DMEM/F12 (1:1) containing 10% fetal bovine serum (FBS), penicillin G (100 units/ml) and streptomycin (100 μg/ml). Cells were centrifugated at 1000 rpm for 5 min. The pellet was suspended in serum-free medium DMEM/F12 (1:1). The dissociated cells were cultured at a density of 5 × 10^4^ cells/ml in medium DMEM/F12 (1:1) supplemented with B27, 10 ng/ml bFGF and 10 ng/ml EGF, at 37 °C in a humidified 5% CO_2_ incubator. Within 5–7 days, cells grew as free floating neurospheres that were then collected by centrifugation, and chemically and mechanically dissociated to obtain a new passage. For cells differentiation, neurospheres were chemically and mechanically dissociated. After counting, 2.5 10^5^ cells were plated on poly-D-lysine (PDL) (10 µg/ml)-coated 24 well plates, or 5 × 10^4^ cells were plated on PDL (10 µg/ml)-coated 96 well plates in medium DMEM/F12 (1:1) supplemented with B27. After 2 hours, cells were treated with different lipids.

### Primary cell culture

Embryonic brains were isolated from E13 timed pregnant mice. The lateral portion of the dorsal telencephalon was dissected and dissociated as previously described^18^. 5 × 10^5^ cells were plated on PDL (10 µg/ml)-coated 24 well plates in DMEM/F12 (1:1) supplemented with B27.

### Liposomes preparation, lipids supplementation and fate

Concentrated lipid stocks were prepared as previously described^15^. Briefly, pure lipids were diluted in chloroform and dried in acid-washed glass centrifuge tubes under a stream of nitrogen. Phospholipid samples were suspended at 2–6 mM in phosphate-buffered saline at pH 7.2 and sonicated twice for 5 min at power setting 0.2–0.5% amplitude. All samples were sterilized with 0.22 µm-pore filters (Sartorius). The recovery of phospholipids after filtration was typically 90% or more. The Dynamic light scattering (DLS) analysis revealed and overage diameter of 127 ± 18 nm for Ptdcho and 82 ± 27 nm for PtdEtn liposomes (Supplementary Fig. 5). In addition, we have evaluated by thin layer chromatography (TLC) that the major lipid present in the filtrated solution are liposome-containing phospholipids, and thus, discarding the presence of phospholipids-hydrolyzed species like lysophospholipids (Supplementary Fig. 5). Diluted phospholipids were added to the growth medium at different concentrations, as described throughout the text. The fate of liposome was evaluated by measuring the incorporation of red fluorescence in cell treated with liposome-labeling with Vybrant™ DiI Cell-Labeling Solution (Thermo Fisher) (Supplementary Fig. 5).

### Immunofluorescence

Cells were cultured on PDL (10 µg/ml)-coated glass coverslips 24 well plates in 0.5 ml of media. 1, 3 or 7 days later, cells were fixed with 4% (w/v) paraformaldehyde-sucrose for 30 min at room temperature, permeabilized with 0.2% Triton × 100 and incubated 1 h with 5% BSA. Cell cultures were incubated with the primary antibody overnight at 4 °C followed by incubation with the fluorescent secondary antibody during 1 h at room temperature. Primary antibodies: rabbit anti-βIII-tubulin (1:1000), rabbit anti-GFAP (1:1000), rabbit anti-Olig2 (1:200), mouse anti-MAP2 (1:100) and chicken anti-Nestin (1:1000). Secondary antibodies were: anti-rabbit Alexa Fluor® 488-labeled (1:1000), anti-rabbit Alexa Fluor® 633-labeled (1:100), anti-mouse Cy3-labeled (1:100), anti-rabbit Cy3-labeled (1:1000) and anti-chicken Alexa Fluor® 488-labeled (1:100). To visualize nuclei, cells were stained and then mounted with ProLong^®^ Gold antifade reagent containing DAPI (Molecular probes, Life technologies). Microscopic analysis were carried out using confocal microscope (Zeiss LSM 880) or the Nikon Model Eclipse 800 microscope and quantitative analyzes were performed with Nikon EZ-C1 3.70 Free Viewer Software or Zen image acquisition software (Carl Zeiss). Cells were counted from twenty random fields per well for each individual experiment. At least three independent experiments were performed. The percentages of progenitors, neuronal and glia cell population were calculated against the DAPI-positive total cell number which include undifferentiated stem cells and differentiated neurons and glia cells.

### Western blot analysis

For western blot analysis, neurosphere-derived cells were plated at a density of 2.5 × 10^5^ and cultured on PDL-coated 24 well plates in 0.5 ml media in differentiation conditions. After 2 h, lipids were added. 3 days later, cells were collected, suspended in lysis buffer (50 mM Tris-HCl pH 8.0, 50 mM KCl, 10 mM EDTA, Nonidet P-40 1%, 20 mM NaF, 1 mM Na_3_VO_4_, 1 mM PMSF and 1:1000 protease inhibitor cocktail) and sonicated five times for 5 s at 5% amplitude (Sonics and Materials Inc–Vibra CellTM). For p-CREB, γ-Tubulin, ERK_1/2_ and p-ERK_1/2_ immunobloting, cells were incubated with lipids immediately after plating and collected 5 minutes later for p-ERK or 1 h for p-CREB. Proteins concentrations were determined using bovine serum albumin (BSA) as standard protein and “Pierce^TM^ BCA Protein Assay Kit (Thermo Scientific)” reagent^55^. 10 µg of cell lysate were resolved on 12% SDS-polyacrilamide gel electrophoresis (PAGE) and transferred to a nitrocellulose membrane (Amersham, GE Healthcare). After blocking overnight with 5% nonfat milk in 0.1% Tween TBS and washing, blots were incubated with rabbit anti-βIII-Tubulin for 1 hour (1:4000), or with anti-pERK (1:500), anti-pCREB (1:1000), anti PCNA (1/15000) or anti-ERK (1:500) during overnight at 4 °C. Peroxidise-conjugated anti-rabbit IgG (1:10000, Jackson Immuno Research) was used as secondary antibody. Loading protein control was demonstrated by measuring the levels of γ-Tubulin using anti-γ-Tubulin (1:6000) and developed with secondary antibody peroxidase-conjugated anti-mouse IgG (1:10000, Jackson Immuno Research). Labeled proteins were detected with chemiluminescence reagents (Amersham^TM^ ECL^TM^ Prime Western Blotting Detection Reagent, GE Healthcare).

### MTT assay

For MTT assay, 5 × 10^4^ neurosphere-derived cells were cultured on PDL (10 µg/ml)-coated 96 well plates in 0.2 ml media in differentiation conditions in 96-well plates. 3 or 7 days later, cells were proceed according^22^.

### Cytotoxicity assay

To evaluate cytotoxicity, LDH released from neurosphere-derived cells was assayed using LDH-P UV AA kit (Wiener lab, Rosario, Argentina) according to the manufacturer’s protocol. neurosphere-derived cells (5 × 10^4^ cells in 0.2 ml media) were cultured on PDL (10 µg/ml)-coated 96 well plates in differentiation conditions and treated with different lipids for 3 days. 50 µL of supernatant was collected from the culture, transferred to another 96-well plate, and 200 µl of substrate solution was added. The absorbance was measured at 340 nm every 30 seconds for 3 minutes using a plate reader. The final data were expressed as LDH (U/L).

### 5-bromo-2′-deoxyuridine assay

For 5-bromo-2′-deoxyuridine (BrdU) assay, 2.5 × 10^5^ were cultured on PDL (10 µg/ml)-coated glass coverslips 24 well plates in 0.5 ml media in differentiation conditions. 2 h later, 10 µM of BrdU was added. After 3 days, cells were processed for immunohistochemistry as described above. Mouse anti-BrdU was used as primary antibody and anti-mouse Cy3-labeled as secondary antibody. The percentages of dividing cells were calculated against the DAPI-positive total cell number.

### Time-lapse video microscopy

Mode of cell division, number of dividing cells, and cell survival were analyzed by time-lapse video microscopy^56^. Briefly, neurosphere-derived cells cultures were imaged every 10 min using a Cell Observer microscope (Zeiss) with Axiovision Rel. 4.5 software (Zeiss) and an AxioCam HRm camera. Images were assembled into a movie using the software Timm’s Tracking Tool-TTT^17^, allowing the identification and tracking of individual clones. Cell survival was quantified every 12 h for each condition. Briefly, the number of cells alive at 12, 24, 36, 48, 60, and 72 h was divided by the total number of cells generated before these time-points. The identity of the progeny generated at the end of the time-lapse sequence was determined by post-imaging immunofluorescence staining. The primary antibodies were: mouse anti-MAP2 and rabbit anti-GFAP; secondary antibodies were anti-rabbit Alexa Fluor® 488-labeled and anti-mouse Cy3-labeled.

### Statistical analysis

Statistical analyses were performed using the software GraphPad Prism version 5. Data in the graphics are presented as Mean ± Standard Error of the Mean (SEM) and represent at least three independent experiments. For statistical significance we considered *p < 0.05, **p < 0.01 and ***p < 0.001, using t-test and One-Way ANOVA with appropriate post hoc tests.

## Electronic supplementary material

Supplementary figure and legend

Supplementary video
