# Author Correction: Specific Phospholipids Regulate the Acquisition of Neuronal and Astroglial Identities in Post-Mitotic Cells

**DOI:** 10.1038/s41598-019-55787-3

**Published:** 2019-12-24

**Authors:** Aneley Montaner, Themis Taynah da Silva Santana, Timm Schroeder, Marcelo Einicker-Lamas, Javier Girardini, Marcos Romualdo Costa, Claudia Banchio

**Affiliations:** 1 Instituto de Biología Molecular y Celular de Rosario (IBR, CONICET) Ocampo y Esmeralda, Predio CONICET and Departamento de Ciencias Biológicas, Facultad de Ciencias Bioquímicas y Farmacéuticas, Universidad Nacional de Rosario, 2000 Rosario, Argentina; 2 Brain Institute, Federal University of Rio Grande do Norte, Av. Nascimento de Castro 2155, 59056-450 Natal, Brazil; 3 Department of Biosystems Science and Engineering, Cell Systems Dynamics, ETH Zurich, Basel, Switzerland; 4 Instituto de Biofísica Carlos Chagas Filho, Universidade Federal do Rio de Janeiro, 21949–902 Ilha do Fundão, Rio de Janeiro Brazil

Correction to: *Scientific Reports* 10.1038/s41598-017-18700-4, published online 11 January 2018

The original version of this Article contained a typographical error in the spelling of the author Marcelo Einicker-Lamas, which was incorrectly given as Marcelo Einiker-Lamas. This has now been corrected in the PDF and HTML versions of the Article, and in the accompanying Supplementary Information file.

