# Supplementary material for: Specific Phospholipids Regulate the Acquisition of Neuronal and Astroglial Identities in Post-Mitotic Cells
Source: Sci Rep. 2018 Jan 11;8:460. doi: 10.1038/s41598-017-18700-4 (PMC5765016; doi:10.1038/s41598-017-18700-4)
Supplement: Supplementary file 1 — Supplementary figure and legend [file 41598_2017_18700_MOESM1_ESM.docx]

**SPECIFIC PHOSPHOLIPIDS REGULATE THE ACQUISITION OF NEURONAL AND ASTROGLIAL IDENTITIES IN POST-MITOTIC CELLS**

Aneley Montaner^1^, Themis Taynah da Silva Santana^2^, Timm Schroeder^3^, Marcelo Einicker-Lamas^4^, Javier Girardini^1^, Marcos Romualdo Costa^2^ and Claudia Banchio^1*^

**
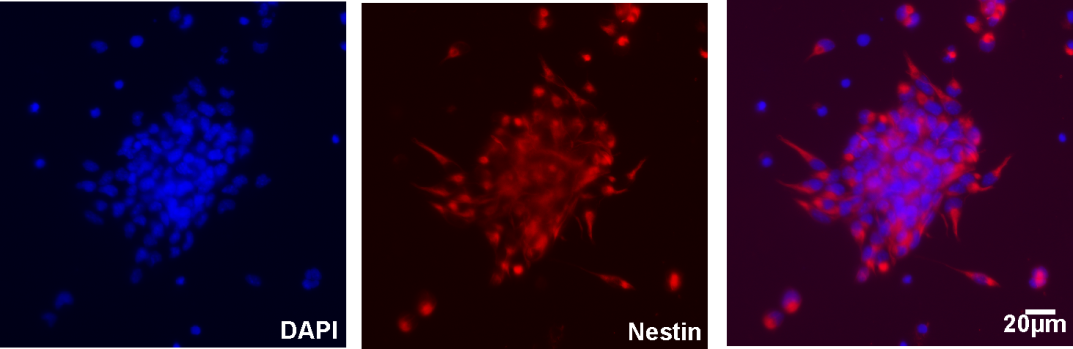
**

**Supplementary Fig. S1:** Immunofluorescence of neurospheres cultured under proliferation conditions. Nuclei (DAPI-blue) and Nestin (red)**.**

**
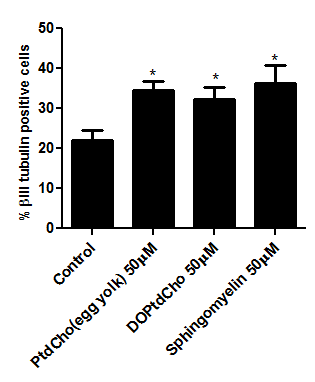
**

**Supplementary Fig. S2:** Effect of sonicated liposomes of dioleyl-PtdCho (DO-PtdCho), egg source-PtdCho and sphingomielyn on differentiation levels.

**

**

**Supplementary Fig. S3:** (A) Lineage trees of progenitor cells showing mode of cell division reconstructed from time-lapse recording. (B) Quantification of mode of cell division after treatment with PtdCho or PtdEtn.

**

**

**Supplementary Fig. S4:** Quantification of neuronal population (cells expressing βIII-tubulin plus Nestin) of primary culture of E13 cortical cells treated with PtdCho for 1, 5 and 24h, fixed and analyzed by immunofluorescence. ***p<0.01.*

**
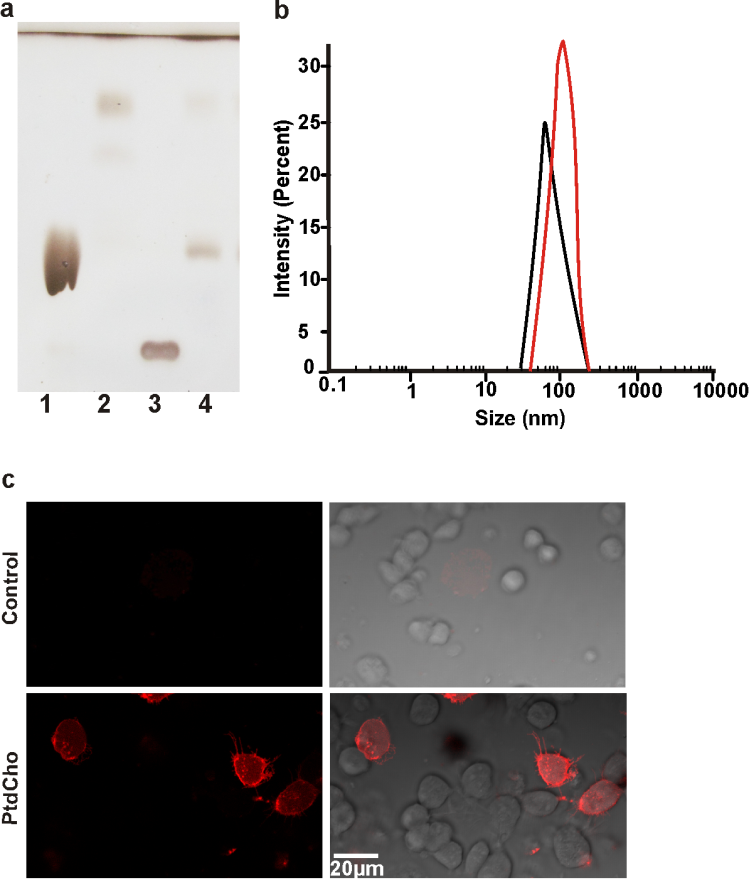
**

**Supplementary Fig. S5: a-** TLC analysis of sonicated phospholipids. The indicated samples were identified by thin-layer chromatography of organic phase on Silica Gel H layers developed in chloroform//methanol/acetic acid/water (50:2.5:8:4, v/v/v/v). Plates were sprayed uniformly with 10% cupric sulfate in 8% aqueous phosphoric acid, allowed to dry 10 min at room temperature, and then placed into a 145 °C oven for 10 min. Each phospholipids was identified by the Rf as described: Rf PtdEtn: 0.81, Rf Ptdcho: 0.31, Rf LPtdcho: 0.15. Line 1: PtdCho standard, Line 2: Liposome of PtdEtn , Line 3: LPtdCho standar and Line 4: Liposome of PtdCho. **b-** Dynamic light scattering (DLS) analysis revealed and overage diameter of 127±18 nm for PtdCho liposomes (red line) and 82±27 nm for PtdEtn liposomes (black line). **c-** Liposome were labeled with Vybrant™ DiI Cell-Labeling Solution (Thermo Fisher) (1:9) and the fate was evaluated by measuring red fluorescence in the confocal microscope (Zeiss LSM 880) and qualitative analyzes were performed with Zen image acquisition software (Carl Zeiss).

**Supplementary Movie**: Real-time observation of cell division and identification of cell fates using post-imaging immunofluorescence. Movie shows the last 20h of observation of a neurosphere-derived cell culture under differentiation conditions (see also Figure 2). The blue arrow points to a post-mitotic astrocyte (GFAP^+^) and the red arrow to a post-mitotic neuron (MAP2^+^). The green arrow points to a progenitor cell dividing and generating two neurons (red arrowheads, MAP2^+^).
